# Erdi Bazhen decoction for cancer-related fatigue due to qi and blood deficiency in prostate cancer patients receiving androgen deprivation therapy: an exploratory randomized, double-blind, placebo-controlled pilot clinical trial protocol

**DOI:** 10.3389/fmed.2026.1970591

**Published:** 2026-10-13

**Authors:** Jintao Wei, Jian Ma, Ziyang Ma, Yang Liu, Lei Xu, Xuefeng Mei, Peihai Zhang

**Affiliations:** 1 The Urology and Andrology Department, Hospital of Chengdu University of Traditional Chinese Medicine, Chengdu, Sichuan, China; 2 Department of Urology, Deyang Hospital Affiliated Hospital of Chengdu University of Traditional Chinese Medicine, Deyang, Sichuan, China

**Keywords:** androgen deprivation therapy (ADT), cancer-related fatigue (CRF), prostate cancer, study protocol, traditional Chinese medicine (TCM)

## Abstract

**Background:**

Prostate cancer is the most common male genitourinary malignancy in China. Long-term androgen deprivation therapy (ADT) causes severe cancer-related fatigue (CRF), which impairs patients’ physical function and quality of life. Currently, there is a lack of safe, long-term specific pharmacological agents for this condition in Western medicine. From the perspective of traditional Chinese medicine (TCM), qi and blood deficiency is the core pathogenesis underlying the occurrence and progression of CRF. Erdi Bazhen Decoction (EBD) has shown favorable clinical effects on fatigue after ADT in routine practice, but high-quality clinical evidence remains scarce, and key parameters for large-scale confirmatory trials remain undetermined. This exploratory pilot trial aims to investigate the preliminary efficacy and safety of EBD granules for CRF in prostate cancer patients receiving ADT, and to estimate critical trial parameters including effect size, recruitment rate, adherence and dropout rate to inform the design of a future large-scale confirmatory RCT.

**Methods:**

This single-center, prospective, randomized, double-blind, placebo-controlled parallel pilot trial will enroll 50 eligible patients from a single hospital. Participants will be randomly assigned to two groups at a 1:1 ratio. Both groups will receive routine supportive care for 4 weeks. The intervention group will receive EBD granules, and the control group will receive matched placebo granules. The primary exploratory efficacy endpoint is the change in total score of the revised Piper Fatigue Scale (PFS-R) from baseline to week 4. Secondary outcomes include quality of life, physical performance and TCM syndrome scores; hemoglobin and C-reactive protein levels will be assessed as exploratory biomarkers. Trial feasibility parameters such as recruitment rate, medication adherence and dropout rate will also be descriptively analyzed to inform future trial design. Safety indicators and adverse events will be fully recorded. Variable block randomization without stratification and full double-blinding will be implemented. The primary analysis will be conducted in the intention-to-treat population; a per-protocol analysis will be performed as a sensitivity analysis. All efficacy estimates are exploratory. This protocol was designed in accordance with the SPIRIT 2025 statement; trial results will be reported in accordance with the CONSORT 2025 statement.

**Conclusion:**

This standardized pilot controlled trial will provide preliminary clinical evidence and essential methodological parameters for EBD in relieving ADT-related fatigue. The results may offer preliminary support for a safe TCM regimen to improve patients’ fatigue, physical status and quality of life, and lay a methodological foundation for integrated Chinese and Western medicine management of prostate cancer-related fatigue. As an exploratory pilot study, the single-center design, small sample size and short intervention period limit the generalizability of confirmatory conclusions. Further large-scale, multi-center, long-term confirmatory trials are needed to verify the long-term effects and population applicability of EBD.

**Clinical Trial Registration:**

https://itmctr.ccebtcm.org.cn/, identifier [ITMCTR2026001527].

## Introduction

Prostate cancer (PCa) is the second most common malignant tumor in men worldwide (1). In China, prostate cancer has become the leading malignant tumor of the male genitourinary system, with its incidence rising with age and predominantly affecting elderly men (2). With the accelerating aging of the Chinese population, PCa has emerged as a major disease that seriously threatens men's health and demands urgent solutions (3).

**Table 1:** Schedule of enrollment, interventions and assessments.

	Enrollment	Allocation	Post-allocation				
Time point			Week 1	Week 2	Week 3	Week 4	Week 5
Enrollment:							
Enrollment screening	X						
Informed consent	X						
Baseline data	X						
Allocation		X					
Intervention:							
Treatment group			X	X	X	X	
Control group			X	X	X	X	
Assessment:							
PFS-R	X					X	
QLQ-C30	X					X	
KPS	X					X	
TCM syndrome score	X					X	
Exploratory biomarkers	X					X	Follow-up
Adverse events			X	X	X	X	Follow-up

Cancer-related fatigue (CRF) is defined as a distressing, persistent, subjective sense of physical, emotional, and/or cognitive tiredness or exhaustion related to cancer or cancer treatment, which is disproportionate to recent activity and interferes with usual functioning (4). CRF is one of the most common treatment-related side effects in prostate cancer, negatively affecting patients’ daily activities, physical, psychological, and functional well-being, and their relationships with others, and impairing quality of life both during treatment and for years thereafter (5). Patients with prostate cancer often require long-term androgen deprivation therapy (ADT) (6). While ADT controls tumor growth, it is frequently accompanied by adverse effects such as fatigue, hot flashes, sleep disturbances, metabolic disorders, and mood changes, making it a key factor in inducing or exacerbating cancer-related fatigue (7). This lends PCa-related CRF distinct disease- and treatment-specific characteristics (8). However, compared with other types of cancer such as breast cancer, research on PCa-related fatigue remains relatively limited.

Currently, modern medicine still lacks a universally recognized specific therapeutic drug for cancer-related fatigue, with clinical management primarily relying on symptomatic treatment (9). Pharmacological interventions are limited by modest efficacy, adverse effects, and restricted applicability, making them insufficient to meet the long-term management needs of patients requiring extended treatment, such as those with prostate cancer (10). Non-pharmacological interventions for CRF have become a research focus in clinical management; however, their efficacy still requires further validation through large-scale randomized controlled trials (11). Psychosocial interventions, such as cognitive behavioral therapy and online psychological counseling, significantly improve patients’ health-related quality of life by alleviating emotional distress and enhancing coping skills (12). In contrast, traditional Chinese medicine (TCM) offers unique advantages and promising prospects in ameliorating cancer-related fatigue, restoring bodily function, and improving quality of life (13). In conclusion, establishing a multidisciplinary comprehensive management model centered on exercise intervention, combined with dietary modification and psychological support, and actively incorporating TCM while conducting localized and individualized intervention studies, will represent an important direction for optimizing PCa-related fatigue management in the future (14).

Qi and blood deficiency, a core concept in TCM, describes insufficient qi activity and depleted blood in the body. Deprived of nourishment from qi and blood, the visceral organs and tissues will develop various debilitating symptoms (15). The classic formula Bazhen Decoction recorded in Zhengti Leiyao is a well-known prescription for the treatment of qi and blood deficiency (16). Erdi Bazhen Decoction (EBD) is a compound Chinese herbal formulation developed by our team based on TCM theory, literature research, and years of clinical experience, modified from Bazhen Decoction. It has been used clinically for many years to ameliorate post-ADT CRF. The formula consists of 11 herbal ingredients, all of which comply with the standards of the Chinese Pharmacopoeia. Modern pharmacological studies have demonstrated that the constituent herbs of this formula, through multi-component and multi-target synergistic actions, target several key pathological processes of post-ADT CRF and exhibit significant activities in regulating immune function, inhibiting tumor proliferation, promoting hematopoiesis, combating fatigue, and improving intestinal function (17).

Although EBD has demonstrated favorable effects in treating post-ADT CRF in clinical practice, high-quality evidence-based data remain scarce. Critically, no prior study has systematically estimated key trial parameters such as effect size, recruitment rate, and adherence for this specific population, which creates substantial design uncertainty for large-scale confirmatory trials. To address this gap, we conducted this exploratory randomized, double-blind, placebo-controlled pilot trial with two primary objectives: first, to explore the preliminary efficacy and safety of EBD in patients with post-ADT CRF; second, to evaluate trial feasibility and estimate critical design parameters including effect size, standard deviation of outcome measures, recruitment rate, medication adherence, and dropout rate. The findings of this pilot study will provide a robust methodological basis for the design and sample size calculation of a future large-scale, multi-center confirmatory RCT, and offer preliminary evidence-based support for improving treatment tolerance and quality of life in this patient population.

## Methods

### Study design

The study is designed as a single-center, prospective, randomized, double-blind, placebo-controlled exploratory pilot clinical trial with two parallel groups. A total of 50 prostate cancer patients with post-ADT CRF and TCM syndrome differentiation of qi and blood deficiency will be recruited from the Department of Urology and Andrology, Hospital of Chengdu University of Traditional Chinese Medicine between 30th September 2026 and 30th December 2026. Participants will be randomized in a 1:1 ratio to either the experimental group (receiving EBD granules plus routine supportive care) or the control group (receiving matched placebo granules plus routine supportive care). The primary exploratory efficacy endpoint is the change in total score of the revised Piper Fatigue Scale (PFS-R) from baseline to the end of the 4-week intervention period. Secondary efficacy outcomes include EORTC QLQ-C30, KPS and TCM syndrome scores. Hemoglobin and C-reactive protein will be evaluated as exploratory biomarkers, as specified in the “Exploratory Biomarkers” subsection below. In addition, process indicators related to trial implementation will be collected and analyzed to evaluate study feasibility and estimate key methodological parameters for future large-scale confirmatory trials. Safety indicators and adverse events will be fully recorded throughout the study. This protocol was designed in accordance with the SPIRIT 2025 statement (18); trial results will be reported in accordance with the CONSORT 2025 statement (19). The study flowchart is presented in Figure 1 and Table 1.

**Figure 1:**
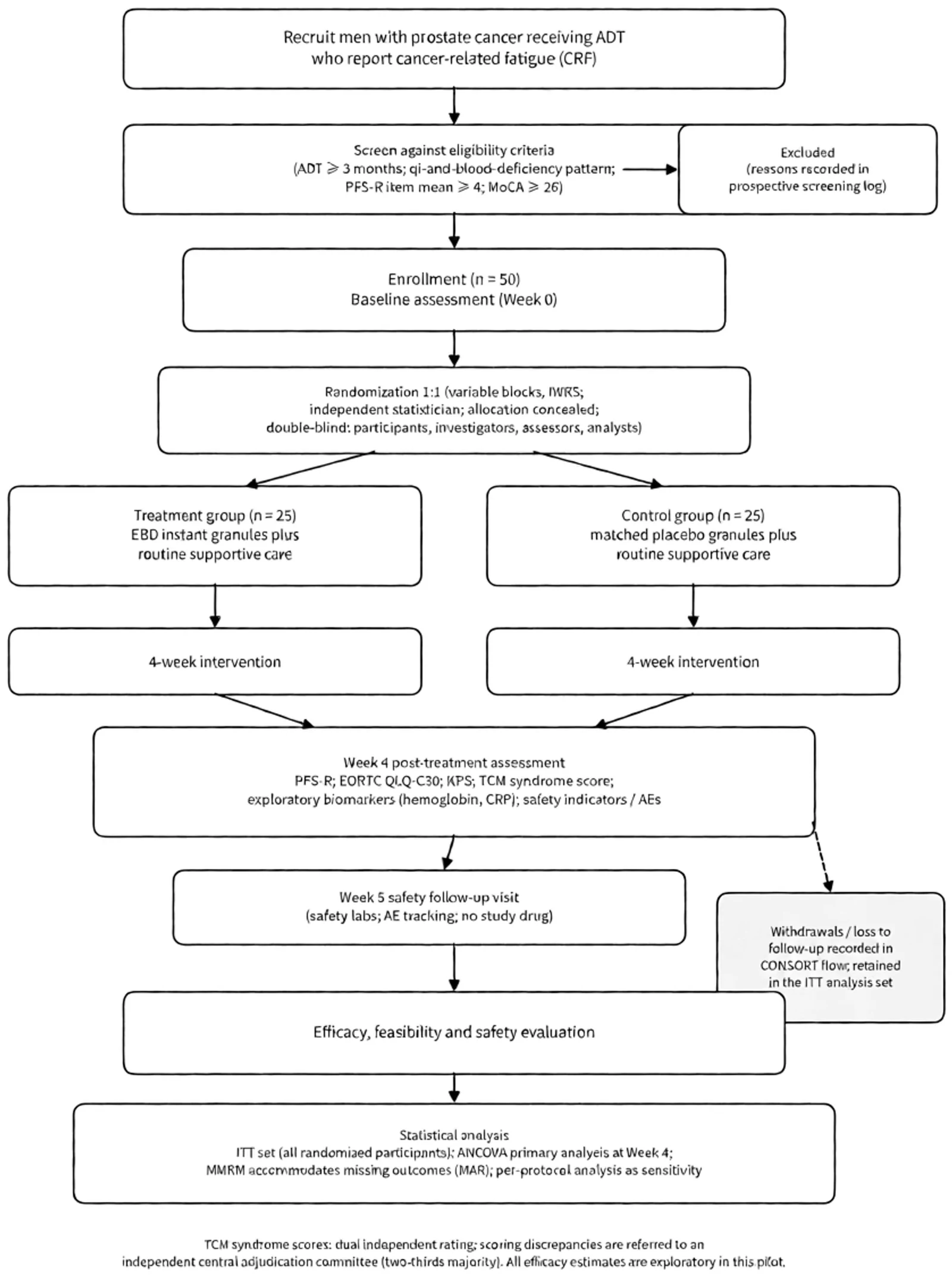
The flowchart of the study protocol.

### Ethics

The study protocol was approved by the Ethics Committee of Hospital of Chengdu University of Traditional Chinese Medicine (approval number: 2026KL-106). The trial was registered at the International Traditional Medicine Clinical Trial Registration Center (ITMCTR2026001527) on 26th May, 2026. The trial complies with the principles of the Declaration of Helsinki and the principles of good clinical practice. The research team declares no financial or non-financial conflicts of interest related to this trial. No commercial pharmaceutical institutions provide funding or economic benefits to researchers; the hospital pharmacy supplies study drugs without the right to interfere with trial implementation and data analysis. The study medication, including both the active EBD granules and the matched placebo, will be prepared, packaged and quality-controlled in-house by the Smart Pharmacy of the sponsoring hospital; the third-party assessment of placebo similarity will be conducted independently of the Smart Pharmacy and of the investigators involved in recruitment, allocation and outcome assessment. All researchers have no patents, equity or other proprietary interests in the study formula. All recruitment and trial procedures will take place at this single site; the affiliation of one co-author with Deyang Hospital does not constitute a recruiting site. As of 22nd September, 2026, the trial has not yet started recruiting. Recruitment is planned to commence on 30th September, 2026 and is expected to complete by 30th December, 2026. Any protocol amendments will be submitted to the Ethics Committee for approval before implementation; approved amendments will be updated in the trial registration record (ITMCTR2026001527) and communicated in writing to all investigators and the Smart Pharmacy, and revised informed consent will be obtained from participants where required.

### Recruitment and participants

Recruitment will be conducted at the Department of Urology and Andrology, Hospital of Chengdu University of Traditional Chinese Medicine. Participants will be screened from outpatient and in-hospital PCa patients via clinical routine consultation. All eligible subjects must strictly meet the unified inclusion and exclusion criteria and voluntarily sign written informed consent before randomization. Comprehensive baseline data will be collected from each participant prior to the initiation of 4-week intervention, covering demographic characteristics including age, height, weight, prostate-specific antigen (PSA) level and Gleason score; medical history information such as disease duration, past medical history, drug allergies and all medications used before trial enrollment, as well as relevant diagnostic laboratory indicators. Experienced attending physicians will evaluate subject eligibility, and research staff will maintain regular follow-up contact to improve medication adherence throughout the trial period. At screening, common alternative contributors to fatigue (such as anemia, thyroid dysfunction, mood/depression, sleep disturbance, and pain) will be evaluated and recorded as baseline covariates.

### Inclusion criteria

The inclusion criteria are as follows:

1. Confirmed diagnosis of prostate cancer with CRF consistent with NCCN diagnostic criteria; currently undergoing ADT for at least 3 months, and the average item score of PFS-R is ≥4.
2. Meets TCM diagnostic criteria for Qi and Blood Deficiency
3. Male aged 45–85 years;
4. Expected survival ≥6 months;
5. Conscious, cognitively normal, MoCA ≥26 at screening, able to complete questionnaires;
6. Willing to participate and provide signed informed consent.

### Exclusion criteria

Subjects meeting any of the following criteria will be excluded from the study:

1. Having severe primary diseases of the cardiovascular system, liver (alanine aminotransferase or aspartate aminotransferase ≥3× the upper limit of normal), kidney [estimated glomerular filtration rate (eGFR) ≤ 30 mL/min/1.73 m^2^, or receiving regular dialysis], lung, and hematological system;
2. Known allergy to any ingredient of EBD or the excipients used in the trial;
3. Patients with severe infection;
4. Concurrent presence of other malignant tumors; patients with cured second primary malignancies for ≥5 years may be eligible
5. In the judgment of the investigator, any condition that is inconsistent with the subject’s best interest for participating in the study, or that may impede, restrict, or interfere with the protocol-specified assessments;
6. Legal disability (including blindness, deafness, muteness, intellectual disability) or mental illness.
7. Concurrent participation in another interventional trial.

#### Dropout criteria

During the trial, subjects meeting any of the following conditions will be considered dropouts:

1. Subjects who are lost to follow-up during the trial and are unable to complete the entire trial and relevant assessments;
2. Subjects who voluntarily withdraw from the trial for personal reasons during the trial and are unable to continue the study.

## Intervention

### Conventional care

Both groups will receive conventional symptomatic supportive care, including disease health education, nutritional guidance, and routine psychological support, administered once every 2 weeks for 15–20 min per session, over a 4-week treatment period.

### Treatment group

In addition to conventional care, EBD instant granules will be added. The herbal composition is as follows: Renshen (Panax ginseng) 9 g, Baizhu (Atractylodes macrocephala) 15 g, Fuling (Poria cocos) 15 g, Zhigancao (Glycyrrhiza uralensis, honey-fried) 5 g, Shengdi (Rehmannia glutinosa, unprocessed) 15 g, Shudi (Rehmannia glutinosa, processed) 15 g, Danggui (Angelica sinensis) 15 g, Baishao (Paeonia lactiflora, white peony root) 15 g, Chuanxiong (Ligusticum chuanxiong) 15 g, Danshen (Salvia miltiorrhiza) 15 g, and Huangjing (Polygonatum sibiricum) 15 g. One dose will be taken daily, divided into three administrations (morning, noon, and evening), each dissolved in 200 mL warm water and taken 30 min after meals.

### Control group

In addition to conventional care, the EBD placebo will be added. One dose daily, divided into three administrations (morning, noon, and evening), each dissolved in 200 mL warm water and taken 30 min after meals.

### Preparation and evaluation process of the EBD placebo

The placebo consists of 5% EBD instant granules and 95% excipients; the excipients will include starch, dextrin, xylitol and caramel color. The exact proportions will be specified in the placebo preparation specification issued and documented in the batch manufacturing record, which will be archived and available for audit. A 5% active placebo will be adopted in this trial rather than a pure blank placebo, based on well-recognized methodological considerations in Chinese herbal medicine double-blind trials. Pure water or blank placebo cannot simulate the unique color, smell, bitter taste, and herbal flavor of traditional Chinese medicinal decoctions, which leads to a high risk of unblinding and severe performance bias. At 5% of the daily therapeutic dose, each constituent is administered far below its pharmacologically active threshold and is therefore unlikely to produce clinically relevant pharmacological activity. Any residual effect of the 5% active placebo would, if present, act in the conservative direction by partially diluting the between-group difference, and would not inflate the estimated treatment effect (20). This active placebo design is a standardized and widely accepted blinding strategy in TCM randomized controlled trials to maintain double-blind validity and avoid subjective unblinding (21, 22). The placebo and the active drug will be identically packaged, with each packet having the same weight. The placebo was similar to the active drug in appearance, odor, and texture. After the drug and placebo are prepared, they will be randomly labeled as Drug A and Drug B. Third-party researchers will conduct a manual similarity assessment covering aspects such as appearance, odor, and texture. Each item will be scored on a scale of 1 to 5, ranging from “not similar” to “similar.” A score greater than 3 indicates high similarity between the two evaluated objects. Both the placebo and the treatment group medication will be supplied by the Smart Pharmacy of the Affiliated Hospital of Chengdu University of Traditional Chinese Medicine. All raw herbal materials of them will be purchased from standardized qualified Chinese medicinal material suppliers in Sichuan, China, and were strictly identified and screened according to the Chinese Pharmacopoeia (2020 Edition). The study medication is a no-decoction herbal formula (dispensing granules) prepared by the Smart Pharmacy of the Affiliated Hospital of Chengdu University of Traditional Chinese Medicine. Unified quality control included verification of species and origin by professional pharmacists to ensure correct botanical origin and medicinal parts and to exclude adulterated, deteriorated, sulfur-fumigated, or pesticide-residue-unqualified herbs; use of the same manufacturer and production batch throughout the trial to eliminate batch-to-batch variation; standardized dispensing by the Smart Pharmacy according to a fixed prescription and uniform dosage, with no raw material replacement during the trial; screening for heavy metals, pesticide residues, aflatoxins, and microbial contamination; storage of the prepared granules in a sealed, low-temperature refrigerated environment (2–8 °C) and use within the valid storage period; and systematic archiving of purchase certificates, batch numbers, quality inspection reports, and dispensing records to ensure full traceability throughout the trial. Chemical identification will be further verified by high-performance liquid chromatography fingerprinting of the single trial batch, with batch similarity assessed against the reference fingerprint of the standardized formula. In addition, the contents of two index constituents, ginsenoside Rb1 and paeoniflorin, will be quantitatively determined by HPLC according to the methods specified in the Chinese Pharmacopoeia (2020 Edition) and must meet the Pharmacopoeia acceptance ranges. Fingerprint chromatograms and assay reports will be archived with the batch records and reported in the final trial publication. The placebo will be produced and packaged by the same Smart Pharmacy in a single batch and will undergo the same heavy-metal, pesticide-residue, aflatoxin, and microbial-contamination screening, package-weight checks, and traceability documentation as the active granules; similarity in appearance, odor, and texture will be verified by the third-party assessment described above.

### Randomization, allocation and blinding

This study adopts a randomized, double-blind design. To ensure balanced allocation, central randomization will be carried out by an independent statistician using an interactive web response system (IWRS), with variable block sizes (4 and 6). The randomization will be performed using SAS statistical software and a 1:1 allocation ratio. Drug packages will be labeled with randomization codes, while allocation codes will be sealed and stored separately. Participants will be enrolled sequentially by clinical staff and randomized through the IWRS. Treatment codes will be retained by an independent statistician or the pharmacy, not by the principal investigators. All parties involved in the trial will remain blinded to treatment allocation. Emergency unblinding will be only permitted in the event of a serious adverse event requiring urgent management, as determined by the principal investigator. At the Week 4 assessment, before unblinding, participants and outcome assessors will be asked to guess the assigned treatment. The proportion of correct guesses will be reported and compared with chance as an objective check on the success of blinding.

### Endpoint measurements

#### Primary endpoint

The primary exploratory outcome measure of this pilot trial is the degree of improvement in CRF, assessed using the Piper Fatigue Scale-Revised (PFS-R) (44), an internationally recognized CRF assessment tool that comprehensively captures the multidimensional characteristics of fatigue. The scale consists of 22 items across four dimensions: behavioral, affective, sensory, and cognitive. Each item is scored on a scale of 0 to 10, with higher total and dimensional scores indicating more severe fatigue. Assessments will be conducted at baseline and at the end of the 4-week treatment period. The primary endpoint is the change from baseline in the PFS-R total score at Week 4. All efficacy analyses are exploratory and intended to estimate effect size for future confirmatory trials.

### Secondary endpoints

#### Quality of life indicator

Assessed using the European Organization for Research and Treatment of Cancer Quality of Life Questionnaire Core 30 (EORTC QLQ-C30) (45), which comprises three sections: functional scales, symptom scales, and global health status. Measurements will be conducted before treatment and at the end of the 4-week treatment period (Week 4). The analytic endpoints will be the changes from baseline in each domain score and the total score.

#### Functional status indicator

Evaluated using the Karnofsky Performance Status (KPS) scale (46). This scale assesses patients’ physical performance and self-care ability on a scale of 0 to 100, with higher scores indicating better functional status. Assessments will be performed before treatment and at the end of Week 4. The analytic endpoint will be the change from baseline in the KPS score.

### TCM syndrome score

Based on the scoring criteria specified in the Guiding Principles for Clinical Research of New Chinese Medicines, major TCM symptoms including fatigue and lassitude, shortness of breath and reluctance to speak, sallow complexion, and poor appetite will be graded and quantified. Each symptom will be scored on a 0–6 scale (0 = absent, 2 = mild, 4 = moderate, 6 = severe). All outcome assessments will be performed at baseline and at the end of Week 4, and the change in total TCM syndrome score from baseline will be defined as a secondary analytical endpoint. Strict quality control procedures will be implemented for observer-dependent syndrome evaluation to ensure assessment consistency and reliability. Briefly, all investigators responsible for TCM syndrome scoring will receive unified centralized training and will be required to pass a standardized examination consisting of 10 typical clinical cases. Only investigators achieving a Kappa coefficient and intraclass correlation coefficient (ICC) ≥ 0.80 relative to the gold standard established by senior TCM physicians will be permitted to conduct study assessments.

For each enrolled case, dual independent blinded rating will be performed by two certified investigators to minimize assessment bias. Consistent results from the two raters will be adopted as the final score. In cases of scoring discrepancy that could not be resolved through internal discussion, complete case materials will be uploaded to the data management system within 24 h and will be submitted to an independent central adjudication committee comprising three senior TCM physicians, each with at least 10 years of clinical experience in TCM oncology or cancer-related supportive care. Committee members will have no role in recruitment, treatment delivery or data collection, will be independent of the recruiting team, and will remain blinded to group allocation; final judgments will be made by a two-thirds majority. Furthermore, regular monthly calibration, feedback, and targeted re-training will be conducted to standardize rating behaviors.

### Exploratory biomarkers

Hemoglobin and C-reactive protein will be measured as exploratory biomarkers. Hb will be used to assess changes in anemia status, and C-reactive protein will be used to reflect alterations in systemic inflammation. Venous blood samples will be collected and tested at baseline and at the end of Week 4. Changes from baseline in these two biomarkers will serve as the analytic endpoints.

For hemoglobin analysis, we will restrict the evaluation to participants with baseline hemoglobin below the normal range, prospectively defined as Hb < 130 g/L for adult males. Given the ceiling effect of hemoglobin and no hemoglobin threshold will be required at study entry, patients with normal baseline hemoglobin have limited potential for further improvement. This hemoglobin subgroup analysis is strictly exploratory and will not be used for confirmatory inference.

### Safety outcomes

#### Adverse events

All adverse events occurring during the trial will be recorded, including symptoms, signs, severity, time of onset, duration, management, and outcome. Severity will be graded according to the Common Terminology Criteria for Adverse Events, version 6.0 (CTCAE v6.0) (23). We encourage participants to proactively report any discomfort at any time. The principal investigator will be responsible for grading adverse events and assessing causality. Expectedness will be judged against the known adverse-effect profile of the study medication; suspected unexpected serious adverse reactions (SUSARs) will be reported to the ethics committee and the competent authority within the applicable regulatory timeframe. All serious adverse events and suspected unexpected adverse drug reactions will be confirmed through collective discussion by the research team. All adverse events will be recorded in the adverse event table of the case report form. Serious adverse events (SAEs) will be reported to the ethics committee and the medical affairs department of the institution within 24 h of awareness, while necessary medical measures will be taken to safeguard participant safety. The occurrence of all adverse events will be summarized periodically. Based on the nature and severity of the adverse event, appropriate management measures will be taken. As this is a fixed-dose trial, dose adjustment is not planned; management measures include observation, symptomatic treatment, temporary suspension (3–5 days), and, if necessary, discontinuation of the study drug/trial participation, and the participant will be followed until the event is resolved or stabilized. All management measures will be documented in detail. The safety analysis set will comprise all randomized participants who received at least one dose of the study medication.

#### Laboratory safety indicators

Complete blood count, liver function, renal function, electrocardiogram, prostate-specific antigen level, and testosterone level will be measured before treatment and at the end of the 4-week treatment period. Any abnormalities and their relationship to the study drug will be recorded.

#### Follow-up

The intervention lasts 4 weeks. A dedicated safety follow-up visit is arranged in Week 5 after medication completion. No study drug or placebo will be given thereafter. Researchers will recheck all safety indices, including blood routine, liver and renal function, ECG, PSA and testosterone levels. PSA and testosterone are monitored to track the underlying prostate cancer and androgen-deprivation status rather than study-drug efficacy. A testosterone level above the castrate range or a rising PSA will be reviewed by the treating urologist or oncologist; biochemical progression or castration escape will be reported as an intercurrent event and will trigger the prespecified discontinuation criterion “disease progression requiring a change in the antineoplastic treatment regimen.” The principal investigator, together with the treating oncologist, will act on any abnormal result and arrange referral; the study team will not advise participants to alter ADT. All adverse events will be tracked, with records of symptom changes, recovery time and treatment outcomes until subjects recover fully or vital signs stabilize. Close follow-up will be conducted for subjects with allergies, gastrointestinal intolerance and other AEs. Continuous monitoring and full management records are required for serious adverse events. Multiple attempts will be made to contact drop-out and lost-to-follow-up participants to supplement final safety data.

#### Post-trial care and compensation for harm

After the Week 5 safety follow-up, no further study drug or placebo will be provided, and participants will return to routine urological and oncological care. Any health problem causally related to the study intervention will be treated according to standard clinical practice, and medical costs for trial-related injury will be borne by the hospital. Withdrawal from the study will not affect the participant's routine medical care.

#### Permitted or prohibited concomitant interventions

During the trial, the following medications and supplements will be prohibited because they may affect fatigue assessment or confound efficacy evaluation: (1) systemic corticosteroids, other than short courses prescribed for acute medical conditions, which will be documented; (2) psychostimulants, including methylphenidate and modafinil; (3) erythropoiesis-stimulating agents and blood transfusion; (4) antidepressant treatment initiated after enrollment (stable antidepressant therapy established before enrollment will be permitted and documented); (5) other Chinese herbal preparations with qi- or blood-tonifying indications; and (6) ginseng-containing supplements. Necessary medications for the management of underlying diseases or adverse events are permitted, but the drug name, dosage, time of use, and reason must be documented in detail and will be summarized; any influence of permitted concomitant medication on the primary endpoint will be examined only in a labelled secondary analysis Participants receiving stable-dose anticoagulant or antiplatelet therapy for an established indication will remain eligible and will continue their prescribed therapy; initiation of such therapy during the trial will be permitted when clinically required and will be documented. In this subgroup, prothrombin time and the international normalized ratio will be measured at baseline and at the end of Week 4, and bleeding events (including epistaxis, gingival bleeding, subcutaneous ecchymosis, hematuria, melena and other clinically significant hemorrhage) will be recorded and reported as adverse events of special interest, in view of the blood-activating constituents of the formula.

##### Criteria for discontinuation or modification of the intervention

The intervention will be discontinued immediately if any of the following occurs: (1) a serious adverse event that is assessed by the principal investigator as related to the study medication, or in whom continued administration is considered unsafe; SAEs unrelated to the study medication and not precluding continued participation will not automatically lead to discontinuation; (2) disease progression requiring a change in the antineoplastic treatment regimen; or (3) voluntary withdrawal from the study by the participant. During the trial, the drug dosage will be generally not adjusted. In the event of intolerance (e.g., gastrointestinal reactions), the medication could be temporarily suspended for 3–5 days and resumed after symptom relief, with the duration of and reason for suspension recorded.

#### Compliance monitoring

Medication compliance in the two groups will be assessed by comparing the ratio of the actual administered dose to the prescribed dose. Daily records will be kept for all medications dispensed to patients or not administered. To enhance patient medication compliance, the following measures will be implemented in this trial: 1) A two-week supply of medication will be dispensed at each visit, and compliance will be reinforced through weekly telephone follow-up at the end of each week; 2) Medication diary cards will be issued to record daily medication intake; 3) A WeChat group will be established to send weekly medication reminders.

For the overall quality control and feasibility evaluation of the trial, we will also systematically collect and summarize data on screening success rate, monthly enrollment rate, completion rate of each assessment, and overall dropout rate. These process indicators will be analyzed descriptively to characterize the feasibility of implementation of the trial protocol, and to provide a reference for protocol optimization and resource allocation in subsequent multi-center confirmatory trials.

#### Sample size

This study is designed as an exploratory pilot trial, whose primary objectives are to estimate effect size, evaluate trial feasibility, and provide parameters for subsequent confirmatory trials, rather than to confirm definitive efficacy through hypothesis testing. Therefore, sample size estimation does not follow the power calculation formula for confirmatory RCTs.

In accordance with established methodological recommendations for pilot randomized controlled trials, a sample size of 15–30 participants per group is generally sufficient to estimate treatment effect parameters, assess feasibility outcomes, and characterize variability in primary endpoints. Given the single-center setting and an estimated 10% dropout rate, we set the sample size at 25 participants per group, for a total of 50 participants across both groups (24).

This sample size aligns with standard pilot trial conventions. With *n* = 25 participants per group, we prespecified a target precision corresponding to a half-width of approximately 0.57 for the 95% confidence interval of the standardized effect size (Cohen's d). We acknowledge that this sample size will yield relatively wide 95% CIs for the mean difference, standard deviation, and effect size of the primary endpoint, and thus these estimates will have limited precision. The data generated will support preliminary characterization of recruitment rate, treatment adherence, and dropout rate. The assumed 10% dropout rate is based on our preliminary observational data in this patient population. These preliminary parameters will serve as the core basis for sample size calculation and trial design in the future large-scale confirmatory RCT.

#### Data collection and management

All data will be collected by uniformly trained researchers at designated time points. Baseline data (demographic data, clinical characteristics, prior treatment history, etc.) will be collected at enrollment; outcome indicators will be assessed at the time points specified earlier. To improve data quality, all assessors will participate in uniform training and become familiar with the scale assessment criteria. Raw data will be recorded on uniformly printed case report forms. A dual independent entry method will be adopted, whereby two researchers independently will enter the case report form data into the ResMan database. Consistency comparison will be performed after entry, and any discrepancies will be corrected by referring to the original case report forms. All participants will be assigned a unique study number in the order of enrollment, which does not contain personal identifying information such as names or ID numbers, and this number will be used as the identification code in the database. Paper case report forms will be stored in a locked filing cabinet under the custody of a designated person; the electronic database will be password-protected and accessible only to the study principal investigator and data manager. All data will be used solely for this study and will not be disclosed to third parties. Data will be backed up regularly; after database lock, data could not be modified, and any necessary corrections required approval from the principal investigator and documentation of the reason.

#### Data analysis

All statistical analyses will be performed using R (version 4.3.1). All efficacy analyses are exploratory in nature and will focus on estimating treatment effects and their 95% confidence intervals, rather than on definitive hypothesis testing. *P* values will be reported as descriptive reference only, and no confirmatory causal inferences will be drawn from statistical significance.

All statistical tests in this study will be selected based on normality testing of continuous variables. Specifically, the Shapiro–Wilk test will be adopted for normality verification, with a significance threshold of *p* > 0.05 to determine the normal distribution of data. For data conforming to normal distribution, parametric statistical tests (e.g., independent samples t-test, paired t-test) will be applied. For data violating normal distribution, non-parametric statistical tests (e.g., Mann–Whitney U test, Wilcoxon signed-rank test, Kruskal–Wallis H test) will be used for statistical analysis.

For descriptive statistics, continuous variables conforming to a normal distribution will be expressed as mean ± standard deviation; non-normally distributed variables will be expressed as median (interquartile range). Categorical variables will be expressed as frequencies and percentages. Baseline characteristics of the randomized groups will be reported descriptively in a baseline characteristics table, including the age distribution and the proportion of participants aged 65 years or older. Process indicators of trial implementation, including screening success rate, recruitment rate, medication adherence rate, assessment completion rate and dropout rate, will be summarized using descriptive statistics with corresponding 95% confidence intervals, to evaluate the feasibility of the trial protocol.

The primary endpoint of this study will be defined as the change from baseline in the PFS-R total score at Week 4, and the between-group difference will be assessed using analysis of covariance (ANCOVA) with the treatment group as a fixed factor and the baseline PFS-R total score as a covariate. The primary ANCOVA model is prespecified to include treatment group as a fixed factor and the baseline PFS-R total score as the sole covariate; no post-randomization variables, including concomitant medication use, will be added to the primary model. Any adjustment for permitted concomitant medication will be reported separately as a secondary analysis. Secondary and exploratory outcomes cover within-group changes, QLQ-C30 scores, KPS scores, TCM syndrome scores and biomarker analyses, with within-group alterations analyzed by the paired t-test for normally distributed data or the Wilcoxon signed-rank test for non-normally distributed data. For efficacy comparisons, between-group differences will be examined via the two-independent-samples t-test or Wilcoxon rank-sum test, focusing on the mean difference with its 95% confidence interval and the standardized effect size (Cohen's d). Exploratory subgroup analyses stratified by age, Gleason score and baseline fatigue severity will also be performed to explore potential heterogeneity of treatment effects; however, these analyses are hypothesis-generating only. With 25 participants per group, estimates will be imprecise and will be reported descriptively with 95% confidence intervals; no confirmatory conclusions will be drawn from subgroup findings. All randomized participants are included in the intention-to-treat (ITT) set regardless of treatment received, data completeness, or adverse events, and account for every participant in the CONSORT flow diagram and AE reporting.

The primary approach to missing outcome data will be the mixed-model repeated measures (MMRM) analysis under the missing-at-random assumption, which uses all available observations without imputation. Multiple imputation (20 imputed datasets, Rubin's rules) and a per-protocol analysis will be conducted as sensitivity analyses. As PFS-R is assessed at only two time points, the MMRM is specified as a two-level repeated-measures model with a group-by-time interaction and a compound-symmetry covariance structure to avoid overparameterization at this sample size (25); ANCOVA is the prespecified primary analysis of the Week 4 endpoint, whereas the MMRM is the mechanism by which missing outcomes are accommodated within the primary analysis under the missing-at-random (MAR) assumption, using all available observations without imputation; the MMRM is therefore not an additional sensitivity analysis, and multiple imputation and the per-protocol analysis constitute the sensitivity analyses (26).

#### Data monitoring and interim analysis

This is an investigator-initiated exploratory clinical study with no external sponsor, and therefore no scheduled audits will be conducted. The study is subject to supervision and inspection by the institutional research administration department and the ethics committee, which are responsible for routine oversight, regular verification of data records, protocol compliance and adverse event management. In the event of random inspections organized by superior competent authorities, the investigators shall fully cooperate and provide complete study documents. Auditors are independent of the study implementation team to ensure objective supervision.

No formal interim analysis will be performed in this study. The principal investigator may decide on early trial termination if any of the following circumstances occur:

1. Serious adverse events related to the intervention occur, and the trial is assessed to be unfeasible to continue;
2. External evidence proves the intervention to be ineffective or harmful;
3. Difficulties in participant recruitment prevent achievement of the target sample size.

The final authority for trial termination rests with the principal investigator and the ethics committee. All termination decisions shall be documented in writing with detailed justifications.

No independent dedicated Data Monitoring Committee is established for this trial. The institutional Ethics Committee of Hospital of Chengdu University of Traditional Chinese Medicine shall act on behalf of the DMC to undertake all data safety monitoring responsibilities throughout the trial. All members of the Ethics Committee are fully independent of the research team, without any financial, academic or proprietary conflicts of interest related to this study.

##### Informed consent

The investigator will provide a detailed explanation to each potential participant or their legal guardian regarding the study purpose, procedures, potential benefits and risks, alternative treatment options, and participant rights. Plain and easily understandable language will be used to fully ensure comprehension. After confirming that the participant has fully understood, the participant voluntarily will sign the informed consent form in duplicate (one copy retained by the participant and one archived by the investigator). For individuals unable to read, an independent witness will be required to be present to witness the oral consent process and sign to confirm. This study will collect only blood samples required for routine clinical tests and will not collect additional biological samples for other research. Should future plans involve the use of residual samples for extended research related to this project, a separate ethics application will be submitted, and additional informed consent will be obtained from the participants.

#### Open science arrangements

The full trial protocol and statistical analysis plan are stored in our hospital's internal document system. Qualified researchers may apply to the corresponding author to view the documents for academic use. After study completion, these files will be uploaded to the International Traditional Medicine Clinical Trial Registration Center for public access.

For result dissemination: simplified result summaries will be provided to participants upon request after data analysis. The complete research manuscript will be submitted to peer-reviewed Chinese and English medical journals. All aggregated efficacy and safety data will be updated to the trial registration center within 12 months after the last participant completes the 5-week follow-up. No trial data or research conclusions shall be used for commercial promotion without ethical approval.

#### Effect size basis for future confirmatory trial

Based on the design positioning of this exploratory pilot trial, the primary purpose of the current study is to estimate the preliminary effect size, standard deviation, and dropout rate of EBD for ADT-related cancer-related fatigue.

The confirmatory trial will be powered (two-sided *α* = 0.05, 90% power) to detect a standardized between-group mean difference of Cohen's d = 0.6 in the change from baseline of the PFS-R score, which represents a pre-specified clinically meaningful effect for cancer-related fatigue, rather than the effect size observed in this pilot trial. The standard deviation estimated from this pilot trial will be used for sample size calculation. We acknowledge that the effect size estimate from this pilot with 25 participants per group is associated with considerable uncertainty. To avoid overestimating the effect, the lower bound of the pilot's 95% CI will be used as a conservative reference if it falls below the prespecified target.

#### Progression, continuation and stopping criteria

To determine the feasibility of a future large-scale confirmatory trial, explicit pilot progression criteria will be predefined in this study, including feasibility progression criteria, protocol revision criteria, and trial stopping criteria.

#### Feasibility progression criteria

The trial will be considered feasible and eligible to support a subsequent formal confirmatory RCT only if all of the following prespecified criteria are met: (1) a target accrual of at least 80% of the planned sample (*n* = 50) enrolled by the end of the planned 3-month recruitment period; (2) a dropout rate, defined as the proportion of randomized participants who discontinue or are lost to follow-up before completing the Week 4 assessment, of no more than 15%; (3) medication adherence, defined as the ratio of the actually taken to the prescribed dose, of at least 80%; (4) an acceptable safety profile, defined as no study-drug-related serious adverse events and a controllable incidence of mild adverse events; and (5) maintained integrity of blinding and trial procedures throughout the study. Monthly recruitment pace will be monitored and reported descriptively as a process indicator rather than as a separate stop/go criterion; the projected pace of approximately 17 participants per month is derived arithmetically from enrolling 50 participants over the planned 3-month window. This projection is considered realistic because the Department of Urology and Andrology manages a concentrated and continuously replenished population of men with prostate cancer receiving long-term ADT, among whom cancer-related fatigue is a common reason for seeking integrative care at this traditional Chinese medicine university hospital, and because all consecutive attendees will be screened at routine ADT follow-up visits, As no formal historical screening registry is available, a prospective screening log will be maintained throughout recruitment, recording the numbers of patients screened, eligible, approached, and enrolled, together with the reasons for screen failure; these data will be used to verify the projected recruitment pace against the restrictive eligibility criteria and to calibrate recruitment planning for the confirmatory trial.

#### Protocol revision criteria

If partial feasibility endpoints are not fully met but no safety risks exist, the protocol will be revised before launching the formal trial:

1. Target accrual between 60%–79%: adjust recruitment strategy, expand screening scope, and optimize inclusion/exclusion thresholds.
2. Dropout rate between 16%–25%: optimize follow-up strategy, strengthen patient education, and refine the intervention administration scheme.
3. Minor non-severe safety issues: optimize the dosage monitoring and traditional Chinese medicine quality control procedure.

#### Trial stopping criteria

The pilot trial will be terminated early, and no further confirmatory trial will be conducted if any of the following occurs:

1. Overall target accrual ＜60% after full recruitment efforts, indicating poor clinical feasibility of implementation.
2. Dropout rate＞25%, showing unacceptable patient adherence.
3. Occurrence of study-related severe adverse events or obvious safety hazards.
4. Uncorrectable blinding failure or major protocol deviation affecting data validity.

## Discussion

CRF is a distressing, persistent, subjective sense of physical, emotional, and cognitive tiredness or exhaustion, and it is a common clinical complication associated with cancer or its treatment (27). Prostate cancer is the malignant tumor with the highest incidence in the male genitourinary system in China, and continuous ADT is the cornerstone and standard treatment for this disease (28). Due to the prolonged duration of ADT, patients’ androgen levels are maintained at persistently very low castration levels, which subsequently leads to severe, persistent, and difficult-to-correct cancer-related fatigue (29). The prevalence of cancer-related fatigue among prostate cancer patients ranges from 50% to 90% (30). Persistent cancer-related fatigue accompanying ADT is a major factor that severely impairs the quality of life of prostate cancer patients, causes treatment interruption, and indirectly affects survival time (31). Modern medicine mainly treats persistent cancer-related fatigue after ADT with central nervous system stimulants and corticosteroids; however, these approaches have limitations such as unsatisfactory efficacy, numerous adverse reactions, and obvious adverse effects with long-term use (32). Moreover, there are no clearly guideline-recommended medications for moderate to severe cancer-related fatigue (33). Therefore, it is of great significance to explore treatment regimens that can be used long-term and have high safety profiles.

From the perspective of TCM, qi functions as the vital energy circulating throughout the human body, whereas blood serves as the fundamental substance for bodily physiological nourishment. Qi and blood deficiency is regarded as the core pathogenic mechanism underlying the occurrence and progression of CRF in TCM theory (34). EBD, a formula derived from a classical prescription Bazhen Decoction, which is traditionally applied to treat qi and blood deficiency syndrome in TCM theory, has been demonstrated by modern pharmacological research to exhibit significant activity in regulating bodily functions and intervening in pathological processes through the synergistic effects of multiple components and multiple targets of its constituent medicinal (35). Multiple published studies have reported that modified Bazhen Decoction exerts positive effects in conditions such as colorectal cancer (36), liver cancer (37), primary dysmenorrhea (38), premature ovarian failure (39), myelodysplastic syndrome (40), autoimmune hemolytic anemia (41), acute lymphoblastic leukemia (42), and depression syndrome (43). However, there have been no reports of its application in post-ADT CRF. Based on this, we intend to objectively evaluate the efficacy in alleviating fatigue and the safety of EBD in prostate cancer patients with post-ADT CRF through a randomized, double-blind, placebo-controlled trial.

This exploratory pilot trial has several inherent characteristics that should be acknowledged when interpreting the findings. First, as a single-center pilot study, enrolled patients are primarily from Sichuan Province, China. Regional differences in lifestyle and clinical practice may limit the generalizability of feasibility parameters, and further multi-center validation is warranted. Second, the sample size of 50 participants is determined by pilot trial methodology and is not powered for definitive confirmatory hypothesis testing. All efficacy results represent preliminary treatment signals and effect size estimates, and cannot be interpreted as confirmatory evidence of efficacy. Third, the relatively short 4-week intervention period and limited follow-up restrict the evaluation of long-term sustainability of treatment effects.

The primary value of this study lies in providing the first randomized controlled preliminary data for EBD in the treatment of ADT-related CRF, and in generating reliable effect size and feasibility parameters to support the design of a subsequent large-scale, multi-center confirmatory RCT. In addition, the implementation-related data obtained from this pilot study, including recruitment efficiency, adherence level and dropout characteristics, will also provide practical reference for the protocol design and resource planning of future multi-center trials. Future confirmatory trials with larger sample sizes, multi-center settings, and extended observation periods will be conducted to verify the long-term efficacy and safety of EBD in this patient population.
